# Labor market effects of rehabilitation for patients diagnosed with dizziness – a Danish nationwide register-based cohort study

**DOI:** 10.1007/s00405-024-08871-y

**Published:** 2024-08-04

**Authors:** Emil Severin Tønnesen, Jesper Bo Nielsen, Kim Rose, Jens Højberg Wanscher, Jesper Hvass Schmidt, Jesper Roed Sorensen

**Affiliations:** 1 Research Unit for ORL–Head & Neck Surgery and Audiology, Odense University Hospital & University of Southern Denmark, J.B. Winsløws vej 4, 5000 Odense C, Denmark; 2 Department of Public Health, University of Southern Denmark, Campusvej 55, 5230 Odense M, Denmark

**Keywords:** Dizziness, Rehabilitation, Labor market, Meniere’s disease, Vestibular neuronitis, Registerø-based

## Abstract

**Purpose:**

Both vestibular neuronitis (VN) and Meniere’s disease (MD) have great impact on quality of life and are associated with a significant number of sick leave days absent from work. The aim was to assess labor market participation rate one year after hospital diagnosis of VN and MD and the use of rehabilitation measures.

**Study design:**

Nationwide register-based cohort study including patients with VN (*n* = 1,341) and MD (*n* = 843) and control persons matched in 1:5 with a VN cohort control (*n* = 6,683) and MD cohort control (*n* = 4,209).

**Results:**

Compared to control persons, VN patients were more likely to be single, have higher income, and a higher Charlson comorbidity index score. MD patients had a higher level of education and a higher Charlson index compared to control persons. One year after patients were diagnosed with VN, no significant difference in labor market participation was observed (*p* = 0.88). However, MD patients had a 10.4% reduced probability of possessing a full-time job one year after diagnosis compared to matched control persons (58.1 ± 0.5% vs. 68.5 ± 0.5%, *p* < 0.001). Both VN and MD patients consulted otorhinolaryngologists, general practitioners, and physiotherapists more than control persons both before and after the initial diagnosis (*p* < 0.01). In addition, MD patients also consulted psychologists more frequently before and after diagnosis of the disease (*p* < 0.01).

**Conclusion:**

Intrahospital diagnosed MD increases the risk of leaving the labor market in opposition to VN. Both MD and VN are associated with significant expenses to the Danish health care system from the use of public rehabilitation measures and medical consultations.

## Introduction

Dizziness or vertigo are common and troublesome conditions with suggested lifetime prevalence of 20 to 30% [1, 2]. Both are accountable for 4 to 4.4% of all main complaints in emergency departments [3, 4]. Dizziness and vertigo can be linked to many possible diagnoses from different medical subspecialties such as otorhinolaryngology, cardiology, neurology, ophthalmology, endocrinology, hematology, and psychiatry. In direct cost of care, the symptoms of dizziness and vertigo represents a substantial and rising economic burden with an estimated annual cost of 48.1 billion dollars in the United States [4–8]. The indirect cost from missed labor participation from Meniere’s disease alone was 585 million dollars/year in the UK [9]. Vestibular vertigo is associated with a significant higher number of days with sick leave compared to non-vestibular vertigo [2]. Three months prior to the diagnosis of vestibular vertigo, reduced workload was found in 70% of patients, 63% had one or more days of sick leave (mean 14 days during the previous3 months), and 6% had quit their job entirely [2]. At three and 12 months after the diagnoses, the loss of working days ranged from 13 to 69 days, respectively [10].

Vestibular vertigo includes conditions affecting the vestibular system with Menieres disease (MD) and vestibular neuronitis (VN) being the most common causes and both having a great toll on patient’s quality of life (QoL) [1, 9]. The two diseases are of different nature with VN being characterized by a one-time acute onset of vertigo lasting days-to-weeks, but with a good prognosis for full recovery [1]. In opposite, MD last for years and has multiple unpredictable attacks of vertigo and often progressive symptoms of vestibular and auditory disease between attacks [1, 9]. Medical treatment of the acute vertigo by either intratympanic or systemic steroids and/or medicine for motion sickness are essential in the treatment to reduce the impact of the diseases. However, other treatments, such as physiotherapy and mental health care support, are also under investigation for their potential to rehabilitate physical and mental function, minimize the chronic phase of the vestibular vertigo, and retain labor market participation [11, 12].

Using the Danish National Registries, we seek to investigate the impact of MD and VN on the risk of losing full time job participation in the labor market. As a secondary outcome measure, we seek to explore the utilization of public available rehabilitation measures.

## Study design and methods

This is a nationwide, register-based cohort study following persons with MD or VN using the Danish Healthcare Registries, which covers all Danish citizens having been in contact with the public hospital system. Anonymized data were extracted for patients diagnosed with either MD (ICD-10 code H81.0) or VN (ICD-10 code H81.2) diagnosed from 1st of January 2013 to 31st of December 2017, but with data collected one year prior to and one year after diagnosis. The time-limits were set to allow for a delay in registration codes for reimbursement and to accommodate varying waiting time for health-care professionals. Age limits were set to 30 to 57 years to ensure a natural connection to the labor market. The National Patient Register (NPR) including health care data registered from all patients visits to both public and private healthcare systems were used [13]. This includes information on all diagnoses, date of admission and discharge, and procedures performed [14]. Due to public reimbursement in rehabilitation measures, any missing data from the private sector is expected to be minimal. However, it is important to notice that both diagnoses are also given in the private sector, which our data does not cover. The patients included in our study are therefore expected to include patients with the heaviest burden of disease.

Patients were divided into two groups, according to ICD-10 code and assessed separately. For both patient groups MD and VN, a control group was formed by matching on age and gender in the ratio of 1:5, with control persons randomly chosen from the entire Danish population. Patients and controls were further compared using sociodemographic data including marital status, level of education, and income. To minimize the risk that any competing disease could explain a potential change in labor market participation, and thereby act as a confounder, we also addressed the level of comorbidity for patients and controls. We used the Charlson Comorbidity Index (CCI) [15, 16]. The CCI consists of 19 items, each representing conditions eligible of shortening patient’s lifespan. Each item has a corresponding score of 1–6 depending on the impact on 1-year adjusted mortality. The control persons were extracted with replacements. All data were anonymised and depersonalised by Statistics Denmark.

## Outcome

The primary outcome is change in labor market attendance after the time of diagnosis of MD and VN. The main hypothesis is, that a diagnosis with chronical dizziness from MD or VN increases the risk of not having full time employment. Being in full-time job is described as a binary parameter as working full time in all 13 weeks of the fourth quarter in the year before or the year following the diagnosis. A random date of full-time labor was chosen for the control groups. Transition to pension benefits were considered a permanent retirement from employment.

Secondary outcomes are investigated using data regarding: (1) utilization of specialist care (oto-rhino-laryngologist and general practitioner) before and after the diagnosis, (2) use of publicly supported healthcare services (psychologist, physiotherapist, and psychiatrist), and (3) use of relevant prescription medication (Anatomical therapeutic chemical classification N05A, N06A, N05BA, N05C, R06AD02, and N06DA covering antidepressants, anxiolytics/hypnotics, and antidementia drugs).

### Statistical analysis

A t-test and chi square analyses were used to compare study groups and their matched control groups using Stata 16.1 (StataCorp, USA).

## Results

### Demography and diagnosis

A total of 843 cases of MD and 1,341 cases of VN where each matched with a control group in a ratio of 1:5; MD control group *n* = 4,209 and VN control group *n* = 6,683 (Table 1). There was no statistical difference in age or gender in either the MD or VN groups compared to the control groups.

**Table 1:** Comparison of sociodemographic data and the Charlson comorbidity index for patients diagnosed with Ménière disease (H81.0) and vestibular neuronitis (DH81.2) and the 1:5 matched control group

	Patients with Meniere disease (*N* = 843)	Matched control group (*N* = 4,209)		Patients with vestibular neuronitis (*N* = 1,341)		Matched control group (*N* = 6,683)		
	Mean	Mean	p-value		Mean	mean		p-value
Age (years)	49.4 ± 7.8	49.4 ± 7.8	0.98		49.0 ± 7.7	49.0 ± 7.7		0.94
Male (%)	40.1 ± 0.5	40.1 ± 0.5	1.00		50.1 ± 0.5	50.1 ± 0.5		0.98
Single (%)	21.0 ± 0.4	23.7 ± 0.4	0.09		20.4 ± 0.4	24.3 ± 0.4		< 0.001
Primary education (%)	2.1 ± 0.1	4.0 ± 0.2	0.01		4.3 ± 0.2	3.3 ± 0.2		0.07
Secondary Education (%)	68.2 ± 0.5	65.8 ± 0.5	0.17		66.8 ± 0.5	66.7 ± 0.5		0.91
Medium education (%)	19.6 ± 0.4	18.6 ± 0.4	0.50		16.7 ± 0.4	18.0 ± 0.4		0.26
Higher education (%)	10.1 ± 0.3	11.6 ± 0.3	0.20		12.2 ± 0.3	12.0 ± 0.3		0.89
Income low (%)	23.7 ± 0.4	25.5 ± 0.4	0.27		22.9 ± 0.4	24.0 ± 0.4		0.40
Income mid to low (%)	24.6 ± 0.4	25.1 ± 0.4	0.72		23.9 ± 0.4	26.0 ± 0.4		0.12
Income mid to high (%)	26.6 ± 0.4	26.3 ± 0.4	0.86		23.6 ± 0.4	24.8 ± 0.4		0.36
Income high (%)	25.2 ± 0.4	23.1 ± 0.4	0.19		29.6 ± 0.5	25.3 ± 0.4		0.001
CCI	0.40 ± 0.9	0.29 ± 0.9	0.002		0.39 ± 1.0	0.29 ± 1.0		< 0.001

Education is highest completed education - divided into four groups and displayed as shares in % income is divide into quartiles and displayed as shares in %. Charlson comorbidity index (CCI) is a weighted index of 19 different conditions each weighing from 1 to 6 points based on their individual mortality risk within 1 year

Both patients with MD and VN presented significantly higher, but persistently low CCI scores compared to the control groups (MD: 0.4 ± 0.9 vs. 0.3 ± 1.0, *p* = 0.002 and VN: 0.4 ± 1.0 vs. 0.3 ± 1.0, *p* < 0.01). Fewer patients lived alone in the VN group compared to the control group (20.4 ± 0.4% vs. 24.3 ± 2.4%, *p* < 0.001). No such difference was found in the MD group.

Regarding education, the MD group was less likely to solely have primary education (2.1 ± 0.1% vs. 4.0 ± 0.2%, *p* = 0.01) compared to the control group. Furthermore, there was significantly more patients with high income in the VN group compared to the control group (29.6 ± 0.5% vs. 25.3 ± 0.4%, *p* = 0.001).

### Labor participation and diagnosis

One year before the diagnosis of MD, fewer persons had full time employment with a negative difference of 3.9% (67.2 ± 0.5% vs. 71.1 ± 0.6%, *p* = 0.02) compared to the matched control group (Table 2). However, this difference increased to 10.4% one year after diagnosis (58.1 ± 0.5% vs. 68.5 ± 0.5, *p* < 0.001). Regarding VN, 2.7% more patients had a full-time job than in the control group (73.3 ± 0.5% vs. 70.6 ± 0.5%, *p* = 0.046) one year before diagnosis, (Table 2). No significant difference was found one year after diagnosis (*p* = 0.88). MD and VN patients had lower levels of granted retirement and disability pension than the matched control groups suggesting that the difference in full time employment were not driven by retirement and disability.

**Table 2:** Comparison of patients with Ménière disease (H81.0) and vestibular neuronitis (H81.2) and 1:5 matched control groups according to change in labor market outcome (change in full time work, pension benefits, and risk of death)

				Patients with Meniere disease (*N* = 843)	Matched control group (*N* = 4,209)		Patients with vestibular neuronitis (*N* = 1,341)	Matched control group (*N* = 6,683)	
				Mean	Mean	p-value	Mean	Mean	p-value
Full time work			Before diagnosis (%)	67.0 ± 0.5	71.0 ± 0.5	0.02	73.3 ± 0.4	70.6 ± 0.5	0.046
			After diagnosis (%)	58.1 ± 0.5	68.9 ± 0.5	0.00	69.0 ± 0.5	68.8 ± 0.5	0.88
Retirement			Before diagnosis (%)	1.1 ± 0.1	1.0 ± 0.0	0.85	1.2 ± 0.1	1.1 ± 0.1	0.67
			After diagnosis (%)	2.5 ± 0.2	2.6 ± 0.2	0.81	2.8 ± 0.2	2.7 ± 0.2	0.84
Disability pension			Before diagnosis(%)	8.3 ± 0.3	9.1 ± 0.3	0.47	6.9 ± 0.3	8.7 ± 0.3	0.03
			After diagnosis (%)	8.5 ± 0.3	9.7 ± 0.3	0.30	7.2 ± 0.3	8.9 ± 0.3	0.04
Death after diagnosis				0.2 ± 0.1	0.3 ± 0.1	0.65	0.5 ± 0.1	0.8 ± 0.1	0.18

Labor market outcomes are binary for full time registration before or after diagnosis – indicated as share of group in %. Before equivalent to 1 year before diagnosia. After is equivalent to 1 year after diagnosis. As the control group is not subject to diagnose their index date is a randomly selected date

### Rehabilitation and health care burden

MD patients consulted oto-rhino-laryngologists significantly more than the control group three months before diagnosis (+ 1,445%, *p* < 0.001) and three months after diagnosis (+ 915%, *p* < 0.001). They also consulted general practitioners more before (+ 32%, *p* < 0.001) and after diagnosis (+ 24%, *p* < 0.001). Patients, who were later diagnosed with MD had a significantly higher use of psychologists compared to controls (+ 121%, *p* = 0.003). This difference increased after diagnosis (+ 1,230%, *p* = 0.001). Physiotherapists were also consulted significantly more before (+ 62%, *p* < 0.001) and after diagnosis (+ 52%, *p* = 0.003). There was no difference in use of psychiatrists (*p* = 0.60) and psychotropics (*p* = 0.28) between patients and controls before and after diagnosis (Table 3).

**Table 3:** Comparison of diagnosed patients with ménière disease (H81.0) and a 1:5 matched control group according to healthcare utilization

		Patients with Meniere disease (*N* = 843)	Matched control group (*N* = 4,209)					
		Mean	Mean	Difference (%)	p-value		∆ in % of pre patient	
ORL	Before diagnosis (%)	40.3 ± 0.5	2.6 ± 0.2	1,445	< 0.001			
	After diagnosis (%)	28.9 ± 0.5	2.9 ± 0.2	915	< 0.001		-28.82	
Physiotherapy	Before diagnosis (%)	7.7 ± 0.3	4.8 ± 0.2	61.5	< 0.001			
	After diagnosis (%)	7.5 ± 0.3	4.9 ± 0.3	50.0	0.003		-4.93	
Psychologist	Before diagnosis (%)	2.3 ± 0.2	1.0 ± 0.1	120.5	0.003			
	After diagnosis (%)	2.6 ± 0.2	1.1 ± 0.1	129.9	0.001		10.52	
Psychiatrist	Before diagnosis (%)	0.8 ± 0.1	1.0 ± 0.1	-18.6	0.61			
	After diagnosis (%)	1.2 ± 0.1	1.1 ± 0.1	13.3	0.72		0.40	
General practitioner	Before diagnosis (%)	75.1 ± 0.4	56.8 ± 0.5	32.1	< 0.001			
	After diagnosis (%)	69.4 ± 0.5	56.2 ± 0.5	23.5	< 0.001		-6.73	
Psychotropic drugs	Before diagnosis (%)	9.0 ± 0.3	7.9 ± 0.3	13.9	0.28			
	After diagnosis (%)	9.5 ± 0.3	7.9 ± 0.3	20.0	0.13		5.26	

The healthcare variables are binary with 1 for any number of visits before or after diagnosis – indicated as share of group in %. Before is measured three months before diagnosis and after is measured as three months after diagnosis – indicated as share of group in %. As the control group is not subject to diagnosis their index date is a randomly selected date. Otorhinolaryngologist (ORL).

Compared to control persons, VN patients consulted oto-rhino-laryngologists more frequently three months before diagnoses (+ 185%, *p* < 0.001). This difference increased after diagnosis (564%, *p* < 0.001). General practitioners were also consulted more before (+ 17%, *p* < 0.01) and after diagnosis (+ 23%, *p* < 0.01). Physiotherapist were consulted more before (+ 30%, *p* = 0.03). The difference increased after diagnosis (+ 82.3%, *p* < 0.01). There was no difference in psychologist consultations before diagnosis (*p* = 0.91). However, after diagnosis there was significant difference (+ 109%, *p* = 0.001). Psychiatrists were consulted more before diagnosis (+ 74%, *p* = 0.02). However, there was no difference between patients and controls three months after diagnosis (*p* = 0.15) No difference between patients and controls in use of psychotropic drugs were found (*p* = 0.94) (Table 4).

**Table 4:** Comparison of patients with vestibular neuronitis (H81.2) and a 1:5 matched control group

		Patients with vestibular neuronitis (*N* = 1,341)	Matched control group (*N* = 6,683)							
		Mean	Mean		Difference (%)		p-value		∆ in % of pre patient	
ORL	Before diagnosis (%)	7.7 ± 0.3	2.7 ± 0.2		185.5		0.00			
	After diagnosis (%)	15.9 ± 0.4	2.4 ± 0.2		564.5		0.00		110.69	
Physiotherapy	Before diagnosis (%)	6.1 ± 0.2	4.7 ± 0.2		29.7		0.03			
	After diagnosis (%)	8.4 ± 0.3	4.6 ± 0.2		82.3		0.00		39.27	
Psychologist	Before diagnosis (%)	0.9 ± 0.1	0.9 ± 0.1		-3,2		0.91			
	After diagnosis (%)	1.9 ± 0.1	0.9 ± 0.1		108.6		0.001		116.67	
Psychiatrist	Before diagnosis (%)	1.7 ± 0.1	1.0 ± 0.1		73.7		0.02			
	After diagnosis (%)	1.4 ± 0.1	1.0 ± 0.1		45.3		0.15		-16.52	
General practitioner	Before diagnosis (%)	63.2 ± 0.5	53.8 ± 0.5		17.4		0.00			
	After diagnosis (%)	67.6 ± 0.5	54.9 ± 0.5		23.2		0.00		5.26	
Use of psychotropic drugs	Before diagnosis (%)	8.1 ± 0.3	7.9 ± 3.3		0.26		0.75			
	After diagnosis (%)	8.0 ± 0.3	7.9 ± 3.4		0.06		0.94		-2.39	

The healthcare variables are binary with 1 for any number of visits pre or post diagnosis – indicated as share of group in %. Pre is measured 3 months before diagnosing and post is measured as 3 months after diagnosing – indicated as share of group in %. As the control group is not subject to a diagnosis their index date is a randomly selected date. Otorhinolaryngologist (ORL).

## Discussion

This study’s main objective was to describe the consequences of MD and VN on labor-market attendance. Earlier studies have shown substantial indirect costs of vertigo due to loss of working days, reduced workload or permanent exit from the labor-market [2, 10]. However, the knowledge within this area is sparse and studies differ in how they classify vestibular vertigo into different sub diagnoses as well as regarding which parameters being investigated (change of job, reduced workload, and quit of job). Based on data from the national Danish registries we have shown that being diagnosed with MD has a major impact on connection to labor market one year after diagnosis, whereas VN seem to have no or little effect in the long-term participation. This could be explained by the different nature of the diseases with MD lasting for years with progressive symptoms of vertigo whereas VN often leads to full recovery [1, 9]. Moreover, as neither of these are granted retirements this is not an explanation for the reduced labor market participation for patients with MD.

The study found both VN and MD patients having more comorbidities based on the CCI score compared to the background population. The data cannot explain this association, and the difference is very small and might not be of clinical significance. However, it could reflect the unknown course of the diseases, other little investigated risk factors, or an increased contact with the health care sector in the search of a diagnosis. A large multicenter study found no difference in CCI score between MD and controls in opposition to our study, but it could reflect a different access to healthcare services in Denmark compared to the USA [17]. We were unable to locate any existing studies addressing the CCI score of patients with VN.

Both VN and MD accounts for significant expenses in the Danish healthcare system regarding the use of publicly reimbursed rehabilitation measures three months before as well as three months after diagnosis. MD patients have a higher use of healthcare services before than after the diagnosis, which might reflect a diagnostic delay by patients or private practice, where patients experience multiple attacks of MD before being referred to a hospital-based system for diagnosis and treatment. Otherwise, it could be that patients with MD are receiving relevant treatment initiated by the general practitioners or oto-rhino-laryngologists with private practice before referral to the hospital system for additional treatment [18, 19]. The decline in use of healthcare services such as physiotherapy and ORL after diagnosis might reflect the fact that the evidence regarding use of vestibular rehabilitation as treatment in the acute and chronic phase of MD is inconsistent [20–22].

For VN the highest expenses in rehabilitation measures were seen after diagnosis, especially in the use of ORL and physiotherapy. Two recent systematic reviews assessing the effect of vestibular rehabilitation in patients with VN [11, 12] and found rehabilitation to have a significant positive impact on the quality of life, decreased various self-perceived dizziness scores, and also the number of abnormal vestibular evoked myogenic potentials (VEMP) as a surrogate marker for otholite dysfunction. They recommended immediate initiation of vestibular rehabilitation as first line of treatment after the diagnoses.

Regarding limitations, the use of a binary outcome for measuring labor market attendance might hamper the interpretation of the results, however this reflects the nature of the data in the Danish national registries. In addition, the quality of data is high leading to a certainness in the conclusion of reduced labor participation after MD. Another issue is lack of information about treatment and diagnosis in the private and primary care sector. However, the Danish Healthcare system including the primary care sector is almost exclusively publicly funded and therefore we regard the data as sufficient and any missing data to be minimal. Lastly the follow-up of one year may be too short to conclude any long term effects on labor market attendance especially for a disease such as Meniere disease with a variability on the presentation and progression of the disease.

## Conclusion

MD diagnosed at public hospitals in Denmark increases the risk of leaving the labor market one year after diagnosis compared to age and gender matched controls. Both MD and VN are associated with great expenses to the Danish health care system in use of public rehabilitation and medical consultations. Future research should assess the effect of faster diagnostics and focus on rehabilitation measure to ensure faster recovery. All patients had an age-based natural connection to the labor market, wherefore any measure securing their ability to at least partially participate in the labor force is deemed of positive value to patients themselves and the society.
